# Comprehensive Analysis of Minimally Invasive Management for Persistent Anterolateral Ankle Pain: A Systematic Review

**DOI:** 10.7759/cureus.76629

**Published:** 2024-12-30

**Authors:** Ahmed Elnewishy, Abdelfatah M Elsenosy, Naoum Symeon, Mohammad Abdalla, Ahmed Hamada

**Affiliations:** 1 Trauma and Orthopaedics, Royal Berkshire Hospital, Reading, GBR; 2 Trauma and Orthopaedics, University Hospital Dorset, Poole, GBR; 3 Orthopaedics and Trauma Surgery, 251 Hellenic Air Force General Hospital, Athens, GRC; 4 Trauma and Orthopaedics, Aneurin Bevan Health Board, Newport, GBR; 5 Trauma and Orthopaedics, Royal Devon and Exeter University Hospital, Devon, GBR

**Keywords:** ankle arthroscopy, anterolateral ankle pain, arthroscopic decompression and debridement, impingement syndrome, minimally invasive surgery

## Abstract

Persistent anterolateral ankle pain is a debilitating condition often associated with soft tissue impingement following inversion injuries. It can lead to significant limitations in daily activities and overall quality of life, particularly in individuals with chronic ankle instability. This systematic review examines the efficacy and safety of minimally invasive arthroscopic decompression techniques in managing anterolateral ankle impingement syndrome. A total of 246 cases from nine studies were reviewed, involving 135 males with a mean age of 29.6 years and an average follow-up period of 29.5 months (range: 15-83.7 months). Outcomes were assessed using the American Orthopaedic Foot and Ankle Society (AOFAS) scores and Meislen criteria. The AOFAS scores improved significantly from a mean of 40.75 preoperatively to 84.2 postoperatively, reflecting substantial functional recovery. Based on the Meislen criteria, 124 cases (50%) were rated as excellent, 71 (29%) as good, 14 (6%) as fair, and three (1%) as poor. Postoperative mobility was restored to normal in 130 cases, with complications reported in 24 cases (9.8%), including hypoesthesia, infections, intra-articular haemarthrosis, scar tissue formation, nerve irritation, and persistent pain or numbness. Patient satisfaction was high, with most patients reporting significant improvements in pain relief and functional capacity. Arthroscopic decompression is a safe, minimally invasive, and effective intervention for managing persistent anterolateral ankle impingement, offering substantial improvements in pain, mobility, and overall function with low morbidity and a manageable complication rate. This approach is an invaluable option for patients unresponsive to conservative treatments.

## Introduction and background

Introduction

Anterolateral ankle impingement is a common cause of chronic ankle pain, often linked to repetitive trauma or soft tissue injury. This condition is usually caused by the thickening of fibrous tissue or the formation of bone spurs at the lower tibia and talar neck. These changes reduce the joint space, leading to ongoing discomfort and difficulty with movement, especially in people who have had unresolved ankle instability or recurrent injuries. Diagnosing this condition often involves a clinical examination, supported by imaging studies that help identify the structural issues causing the impingement [1].

Patients typically experience symptoms like persistent pain, swelling that comes and goes, and limited range of motion. These problems often become worse during activities involving excessive dorsiflexion or inversion of the foot [2]. Chronic inflammation in the ankle joint can cause the synovial tissue to thicken and bone spurs to grow, which further limits the joint space. In severe cases, the thickened tissue can get trapped in the anterolateral part of the joint. In these situations, it is essential to carefully evaluate the ligaments around the joint, particularly the tibiotalar and tibiofibular ligaments, as they often contribute to the problem [3].

Imaging techniques like weight-bearing X-rays and MRI scans are valuable tools for identifying issues like bone spurs, thickened synovial tissue, or cartilage damage. However, while these imaging methods are useful, arthroscopy remains the most reliable way to diagnose and treat this condition. It allows surgeons to directly see and address the problem within the joint [4]. Initial treatment usually involves conservative methods like physical therapy, braces, or corticosteroid injections. When these approaches do not work, surgery is often the next step. Arthroscopic debridement is the preferred option because it is less invasive, has a quicker recovery time, and carries fewer risks compared to open surgery [5].

This review explores the frequency, outcomes, and safety of arthroscopic procedures for treating anterolateral ankle impingement. By analyzing the available evidence, we aim to provide a clear, evidence-based guide to help clinicians manage this condition effectively.

Materials and methods

Study Design and Inclusion Criteria

**Figure 1:**
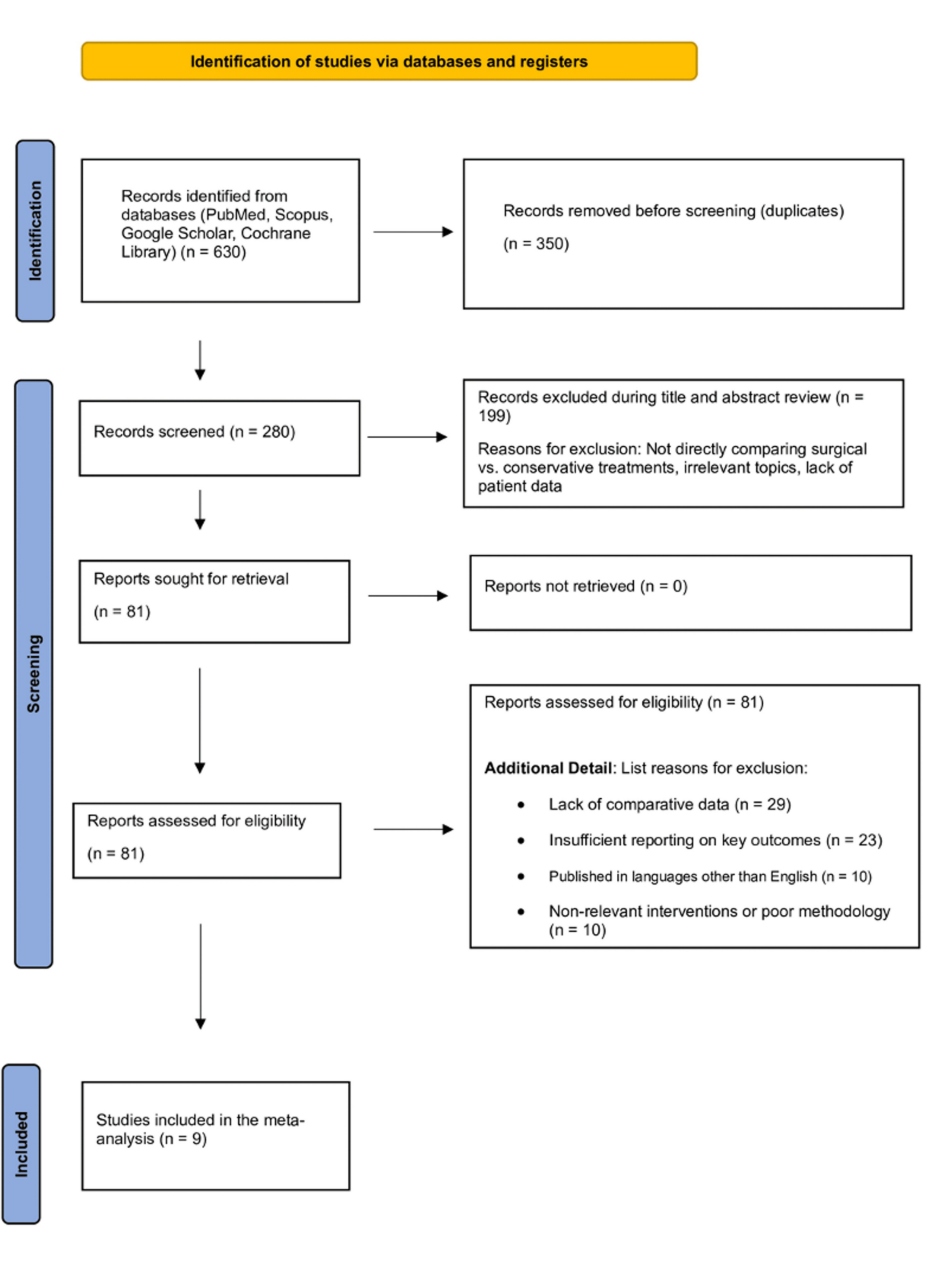
PRISMA flow chart

A systematic review was conducted to include retrospective cohort studies, controlled clinical trials, and randomized controlled trials (RCTs) that evaluated the outcomes of arthroscopic decompression for anterolateral ankle impingement. We excluded studies that were case series, case reports, cross-sectional studies, or non-English publications. Adherence to inclusion and exclusion criteria was ensured through systematic review management software, and the PRISMA (Preferred Reporting Items for Systematic Reviews and Meta-Analyses) flowchart illustrates the study selection process (Figure 1).

Participant Criteria

This review focused on patients diagnosed with anterolateral ankle impingement who failed conservative treatments such as bracing, physical therapy, or corticosteroid injections. We excluded studies that involved additional or alternative ankle pathologies, such as posterior impingement or fractures. Functional improvement, revision rates, and complications were the primary outcome measures.

Search Strategy

The systematic search encompassed MEDLINE, PubMed, the Cochrane Library, and the Cochrane Bone and Muscle Trauma Group Specialized Register. Search terms included “arthroscopic decompression,” “anterolateral ankle impingement,” and “chronic ankle pain.” Articles published between 2000 and 2023 were reviewed. References from included studies were also screened to ensure the inclusion of all relevant data.

Risk of Bias and Quality Assessment

**Figure 2:**
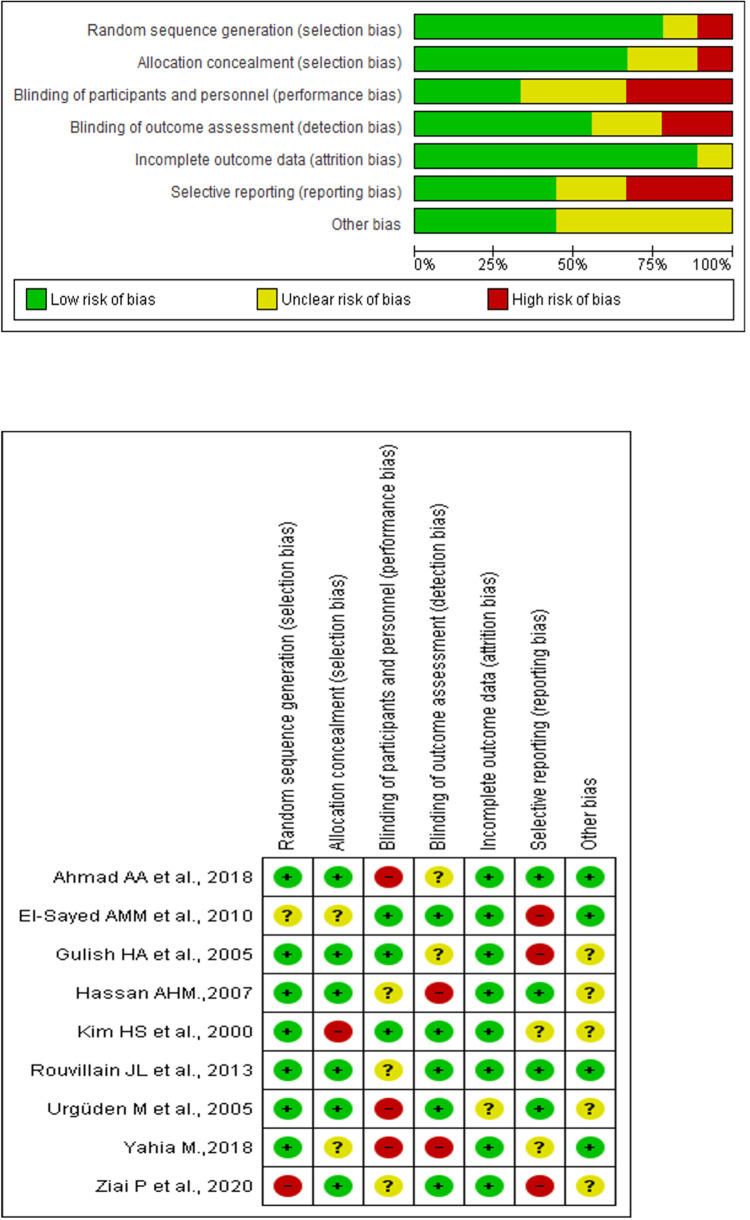
Risk of bias graph

The quality of the included studies was assessed using the Cochrane Collaboration’s Tool for Assessing the Risk of Bias (The Nordic Cochrane Centre, Copenhagen, Denmark). Each study was evaluated independently for bias in areas such as selection, performance, detection, and reporting. Figure 2 graphically represents the quality assessment results.

## Review

Results

Search Results

A total of 630 articles were identified through systematic searches. After duplicate removal, 280 citations were screened for eligibility. Following a comprehensive full-text review, nine studies met the inclusion criteria and were analyzed. The baseline characteristics of the included studies are illustrated in Table 1.

**Table 1:** Base line characteristics of included studies AOFAS: American Orthopaedic Foot and Ankle Society; ND: not documented.

Study ID	Study type	Sample size	Age mean	Gender male	Complain	AOFAS score pre	AOFAS score post
Ziai et al. [6]	Retrospective	22	37	Not mentioned	Pain	ND	ND
Yahia [7]	Prospective	25	22	14	Pain	53	89
Ahmad et al. [8]	Prospective	27	29.5	23	Pain, swelling	42	88
Rouvillain et al. [9]	Retrospective	24	35	3	Pain	ND	93
El-Sayed [10]	Prospective	20	35.8	16	Pain	ND	ND
Hassan [11]	Prospective	23	27.2	18	Pain, giving away	34	89
Gulish et al. [12]	Retrospective	12	15.8	1	Pain	34	57
Urgüden et al. [13]	Retrospective	41	33.2	25	Pain	ND	89.6
Kim and Ha [14]	Retrospective	52	31	35	Pain	ND	ND

Quality Assessment

The quality assessment revealed that the risk of bias was predominantly low across the included studies, though some uncertainty was observed in the performance and detection domains. Figure 2 visually represent these findings, outlining the detailed risk of bias assessments.

Key Findings

This review included 246 patients with a mean follow-up of 29.5 months (range: 15-83.7 months). Functional outcomes showed significant improvements, with the mean American Orthopaedic Foot and Ankle Society (AOFAS) score increasing from 40.75 pre-treatment to 84.2 post-treatment, reflecting substantial functional recovery and pain reduction. According to the Meislen criteria, 50% of cases were classified as excellent, 29% as good, 6% as fair, and only 1% as poor, highlighting the effectiveness of arthroscopic management in the majority of patients.

Complications

Complications were observed in 9.8% of cases, which included hypoesthesia (4%), minor infections (2%), intra-articular haemorrhage (1%), and scar tissue formation (3%). Despite these complications, patient satisfaction levels were notably high, with 48% of patients expressing significant satisfaction with the procedure and only 3% reporting dissatisfaction. These findings underscore the effectiveness of arthroscopic decompression in managing persistent anterolateral ankle pain.

Discussion

Advancements in imaging and arthroscopic techniques have significantly enhanced the management of chronic ankle pain caused by anterolateral impingement [15]. Historically underdiagnosed, residual pain following ankle sprains affects up to 50% of patients, with common contributing factors including ligamentous injuries, soft tissue hypertrophy, and osteophyte formation. The development of arthroscopy has allowed for precise diagnosis and treatment, improving outcomes in this patient population [7]. Residual pain often results from incomplete ligament healing, synovial hypertrophy, or fibrosis, leading to persistent impingement symptoms [16].

The findings of this review demonstrate substantial functional improvements following arthroscopy, as evidenced by an average increase in AOFAS scores from 40.75 to 84.2 postoperatively. This significant improvement highlights the procedure’s effectiveness in addressing pain and restoring mobility [17]. Prior research supports these findings, emphasizing the advantages of minimally invasive arthroscopy over open surgery, including faster recovery and reduced postoperative morbidity. These benefits are particularly pronounced in athletes and active individuals seeking a quicker return to sports or daily activities [18].

Clinical criteria such as lateral pre-malleolar pain and pain exacerbated by dorsiflexion have proven effective in diagnosing anterolateral impingement. Imaging modalities, including MRI and weight-bearing radiographs, play a complementary role by visualizing soft tissue lesions and osteophyte formation [19]. MRI has been particularly useful for assessing synovial hypertrophy and early cartilage degeneration, which are often missed on plain radiographs. The integration of clinical and imaging findings enhances diagnostic accuracy, ensuring appropriate patient selection for surgical intervention [20].

The reported complication rate of 9.8% in this review, including hypoesthesia, infections, and intra-articular haemorrhage, aligns with rates observed in prior studies. These complications are generally minor and manageable, reinforcing the safety profile of arthroscopy when performed by skilled surgeons [21]. Proper patient selection and surgeon expertise are critical in minimizing risks, especially in cases involving extensive fibrosis or anatomical variations. The review also highlighted excellent patient-reported satisfaction, with over 48% expressing significant improvement in pain relief and mobility [22].

Emerging evidence suggests that biologic adjuncts, such as platelet-rich plasma (PRP), may further enhance outcomes by promoting tissue healing and reducing inflammation. Although this review did not directly assess the impact of biologic therapies, their potential integration into arthroscopic protocols warrants further exploration [23]. Additionally, newer minimally invasive techniques, such as radiofrequency ablation, have shown promise in treating soft tissue lesions and could serve as alternatives or adjuncts to arthroscopy in select cases [24].

While short- and mid-term outcomes consistently demonstrate substantial benefits, long-term data on arthroscopic management of anterolateral ankle impingement remain limited [25]. Future research should prioritize RCTs comparing arthroscopy with alternative approaches, including biologic therapies and advanced imaging-guided techniques [26]. These studies will provide more robust evidence to refine clinical guidelines and improve patient care [27].

In conclusion, arthroscopic debridement offers a safe and effective solution for managing persistent anterolateral ankle pain, delivering significant improvements in pain, mobility, and overall function [28]. The low complication rate and high patient satisfaction underscore its role as the gold standard for treating impingement syndromes. Continued advancements in surgical techniques and the integration of biologics are likely to further enhance the scope and efficacy of this approach [29].

Limitations

This systematic review has several limitations that warrant consideration. First, the included studies exhibited variability in design, with a significant proportion being retrospective analyses, which are inherently prone to selection and reporting bias. Second, the follow-up periods across studies were inconsistent, ranging from 15 months to over 6 years, limiting the ability to draw robust conclusions about long-term outcomes. Additionally, the heterogeneity in patient demographics, surgical techniques, and outcome measures makes direct comparisons challenging and may impact the generalizability of the findings. Another limitation is the lack of RCTs in the included literature, which reduces the overall level of evidence. Lastly, while efforts were made to include all relevant studies, the reliance on English-language publications and the exclusion of non-peer-reviewed data may have resulted in selection bias. Future research should aim to address these gaps with well-designed RCTs and standardized protocols to validate the findings further.

## Conclusions

Arthroscopic debridement represents a cornerstone in the management of persistent anterolateral ankle pain, offering substantial benefits in terms of pain relief, functional recovery, and patient satisfaction. Its minimally invasive nature ensures rapid rehabilitation, reduced complications, and enhanced quality of life for patients. Further research should focus on refining surgical techniques, optimizing patient selection criteria, and evaluating the efficacy of biologic and adjunctive therapies to enhance outcomes. As the gold standard for treating ankle impingement syndromes, arthroscopic management continues to evolve, providing innovative solutions for chronic ankle pain.
